# Mapping Visual Field Defects With fMRI – Impact of Approach and Experimental Conditions

**DOI:** 10.3389/fnins.2021.745886

**Published:** 2021-09-08

**Authors:** Gokulraj T. Prabhakaran, Khaldoon O. Al-Nosairy, Claus Tempelmann, Hagen Thieme, Michael B. Hoffmann

**Affiliations:** ^1^Department of Ophthalmology, Otto von Guericke University, Magdeburg, Germany; ^2^Department of Neurology, Otto von Guericke University, Magdeburg, Germany; ^3^Center for Behavioral Brain Sciences, Magdeburg, Germany

**Keywords:** vision, human visual cortex, scotoma, perimetry, retinotopy, glaucoma, retinitis pigmentosa, atlas

## Abstract

Current initiatives to restore vision emphasize the need for objective assessments of visual field (VF) defects as pursued with functional magnetic resonance imaging (fMRI) approaches. Here, we compared population receptive field (pRF) mapping-based VF reconstructions to an fMRI method that uses more robust visual stimulation (on-off block design) in combination with individualized anatomy-driven retinotopic atlas-information (*atlas-based VF*). We investigated participants with sizable peripheral VF-deficits due to advanced glaucoma (*n* = 4) or retinitis pigmentosa (RP; *n* = 2) and controls (*n* = 6) with simulated scotoma. We obtained (1) standard automated perimetry (SAP) data as reference VFs and 3T fMRI data for (2) pRF-mapping [8-direction bar stimulus, fixation color change task] and (3) block-design full-field stimulation [8-direction drifting contrast patterns during (a) passive viewing (PV) and (b) one-back-task (OBT; reporting successions of identical motion directions) to probe the impact of previously reported task-related unspecific visual cortex activations]. Correspondence measures between the SAP and fMRI-based VFs were accuracy, assisted by sensitivity and specificity. We found an accuracy of *pRF-based VF* from V1 in patients [median: 0.62] that was similar to previous reports and increased by adding V2 and V3 to the analysis [0.74]. In comparison to the pRF-based VF, equivalent accuracies were obtained for the atlas-based VF for both PV [0.67] and, unexpectedly, the OBT [0.59], where, however, unspecific cortical activations were reflected by a reduction in sensitivity [0.71 (PV) and 0.35 (OBT)]. In conclusion, in patients with peripheral VF-defects, we demonstrate that previous fMRI procedures to obtain VF-estimates might be enhanced by: (1) pooling V1-V3 to enhance accuracy; (2) reporting sensitivity and specificity measures to increase transparency of the VF-reconstruction metric; (3) applying atlas-based procedures, if *pRF-based VFs* are not available or difficult to obtain; and (4) giving, counter-intuitively, preference to PV. These findings are expected to provide guidance to overcome current limitations of translating fMRI-based methods to a clinical work-up.

## Introduction

Visual field (VF) testing is of critical importance for diagnosis and follow-up in ocular diseases. Standard automated perimetry (SAP) is primarily used for VF-assessment in clinical routine and regarded gold standard. Besides their widespread use, these conventional VF tests suffer from notable limitations. For example, they depend on the participant’s ability and compliance in performing the attentionally demanding subjective test and on the tester’s experience and skill (Gardiner and Demirel, 2008; Junoy Montolio et al., 2012). Such issues have emphasized the need and motivated the development of objective tests which do not require maximal patient compliance.

Interest in this field has been enhanced by current gene- and cell-based initiatives aiming at the restoration of retinal function in ocular diseases (reviews Jutley et al., 2017; Roska and Sahel, 2018), as these benefit from objective readouts of therapy success. Given the recent therapeutic advances at the level of the visual cortex with cortical implants (Beauchamp et al., 2020), one option for an objective VF assessment is the reconstruction of VF-coverage and identification of VF defects from the response patterns in the visual cortex obtained with functional magnetic resonance imaging (fMRI). This approach is based on the retinotopic layout of the visual information in the visual cortex, which can be directly obtained from fMRI data via (i) individualized VF-mapping, e.g., population receptive field (pRF) mapping (Dumoulin and Wandell, 2008), or (ii) indirectly via the application of a group-informed retinotopic atlas to the individual anatomy (Benson et al., 2014). (i) Individualized VF-mapping has been widely applied not only to map and investigate normal visual cortex functioning in healthy individuals (Harvey and Dumoulin, 2011; Wandell and Winawer, 2015; Prabhakaran et al., 2020), but also to provide insights on the interplay of visual cortex stability and plasticity in vision disorders (Baseler et al., 2011; Barton and Brewer, 2015; Hoffmann and Dumoulin, 2015; Ahmadi et al., 2020, 2019). For each voxel in the visual cortex, a model-based analysis of the participant-specific pRF-mapping data is applied to estimate the preferred eccentricity and receptive field size for a population of neurons in that voxel. Subsequently, this can be projected back to the VF for the reconstruction of a VF-map. Previous studies demonstrated a good correspondence of pRF-based VFs with subjective VF-prediction in both patients with VF-defects (Papanikolaou et al., 2014; Silson et al., 2018; Ritter et al., 2019; Carvalho et al., 2021) and healthy individuals with simulated scotomas (Hummer et al., 2018). (ii) For the atlas-based approach, cortical fMRI responses from full-field stimulation (i.e., non-mapping) can be projected into the VF via information from an anatomically driven participant-specific retinotopic atlas. Despite a potential utility of atlas-based VF-predictions, reports addressing this are very limited (Cideciyan et al., 2015) with most studies restricting the use of retinotopic atlases to only delineate visual areas. In fact, the pRF-based approach is intuitively expected to be of highest accuracy. Accordingly, Ritter et al. reported for the pRF-based reconstruction of peripheral VF defects (similar to the present study’s patient cohort) in retinitis pigmentosa (RP) from V1 a median accuracy of 0.85 [range: 0.49–0.98 (*n* = 7)] (Ritter et al., 2019). It should be noted, however, that this approach is subject to the availability of reliable pRF-mapping data and the patient’s reliable fixation of the central fixation target. Importantly, the atlas-based approach is much less dependent on patient’s compliance as it applies more robust visual stimulation in a simple on-off block design. To assess its potential, a direct comparison of pRF- and atlas-based approaches is needed. The present investigation is aiming to fill this gap.

We address the question of how atlas-based and pRF-based reconstructions of VF defects compare for V1 and how they benefit from the inclusion of activity in V2 and V3. We ascertain a quantitative comparison of the different fMRI-based VF predictions to the subjective SAP-derived VFs. Further, the effect of adding stimulus-related attention on atlas-based reconstructions is determined. Finally, we assessed the potential improvement of the VF-reconstruction for a combined pRF- and atlas-based approach [Bayesian Benson (here termed “Combined”): Benson and Winawer, 2018].

## Materials and Methods

### Participants

Individuals with sizable peripheral VF-deficits due to advanced glaucoma (*n* = 4) or RP (*n* = 2). Age of the patients ranged between 46 and 78. One of the RP patients was also diagnosed with secondary glaucoma. One additional participant with RP was excluded on grounds of unreliable mapping data (not included in the above sample size). Healthy controls (HCs) with normal vision [best-corrected decimal visual acuity ≥ 1.0 (Bach, 1996); *n* = 6] were also included for comparisons. Written informed consents and data usage agreements were signed by all participants. The study was conducted in adherence to the tenets of the Declaration of Helsinki and was approved by the ethics committee of the University of Magdeburg.

### Visual Field Testing

Standard automated threshold perimetry (SAP) of the central 30° was performed to measure visual sensitivity using 24-2 Swedish Interactive Threshold Algorithm protocol [Goldmann size III white-on-white stimuli; either: Humphrey Field Analyzer 3 (SITA-Fast); Carl Zeiss Meditec AG; Jena, Germany; or (*n* = 2): OCTOPUS^®^ Perimeter 101, Haag-Streit International, Switzerland; dG2; dynamic strategy]. The SAP-based VFs served as the reference for the correspondence analysis with fMRI-based reconstructions.

### Fixation Stability

An MP-1 microperimeter (Nidek, Padua, Italy) was used in the patient cohort (except GL3) to ascertain the fixation stability of a central fixation target. Fixations were tracked with 25 Hz and the proportion of fixations falling within the central 2° radius was determined using built-in MP1 analysis. All the patients had stability greater than 96%.

### Functional Magnetic Resonance Imaging Measurements

All magnetic resonance imaging (MRI) and fMRI data were collected with a 3 Tesla Siemens Prisma scanner (Erlangen, Germany). One high-resolution whole brain anatomical T1-weighted scan (MPRAGE, 1 mm isotropic voxels, TR | TI | TE = 2500 | 1100 | 2.82 ms) was collected for each participant. fMRI scans parallel to the AC–PC line were acquired using a T2^∗^-weighted BOLD gradient-EPI sequence (TR | TE = 1,500 | 30 ms and voxel size = 2.5^3^ mm^3^). An inversion recovery EPI sequence (TR | TI | TE = 4,000 | 1,100 | 23 ms) with spatial coverage (FOV) and resolution identical to the T2^∗^ EPI was obtained to aid in the alignment of structural and functional data. The visual stimuli for fMRI were generated with Psychtoolbox-3 (Brainard, 1997; Pelli, 1997) in MATLAB (MathWorks, Natick, MA, United States) and back-projected to a screen [resolution: 1,920 × 1,080 pixels] at the rear end of the magnet bore. The visual stimulus was viewed monocularly with the better eye based on SAP [mean deviation (MD) and extent of VF-defect] in the patients and the dominant eye in the controls at a distance of 35 cm via an angled mirror. Only the lower section of a 64-channel head coil was used effectively resulting in a 34-channel coil to allow for an unrestricted view of the entire projection screen. For each participant, we collected in two separate sessions, fMRI data for (1) pRF mapping and (2) block-design full-field stimulation. The block-design data had been analyzed for a previous publication (Prabhakaran et al., 2021), which provided the extraction criteria for the selection of stimulation-driven voxels in our present analysis.

#### Population Receptive Field (pRF) Mapping

##### Visual stimulation

For visual stimulation a moving checkerboard stimulus pattern was presented [directions: 8 (2 horizontal, 2 vertical, and 4 diagonal); mean luminance: 109 cd/m^2^; contrast: 99%; check size: 1.57°), exposed through a bar aperture [width: 1/4th (3.45°) of the stimulus radius (13.8°)]. The bar propagated across a circular aperture spanning the stimulus radius in 16 steps [step rate = 1.75°/repetition time (TR); TR = 1.5 s] within 24 s per bar directions. The sequence of the bar direction alternated with a horizontal or vertical sweep followed by a diagonal sweep, for which only the first 12 s of the sweep were presented and the later 12 s of the sweep were replaced by a mean luminance gray. For the controls, mapping data were obtained with an artificial peripheral (>7°) and complete lower right quadrant scotoma. Each pRF-mapping scan lasted 192 s and was repeated six times for the patient cohort and four times for the controls. The participants responded to a fixation-dot color change via button press.

##### Preprocessing and analysis

Freesurfer^1^ was used for the automated segmentation of gray-white matter boundaries and ITK gray software^2^ for the manual correction of segmentation errors. For each individual participant, within and between-scan head motion artifacts in the fMRI scans were corrected with AFNI^3^ and the motion corrected functional images were aligned spatially to the anatomical scan using Kendrick Kay’s alignment toolbox.^4^ Using MATLAB based Vistasoft tools,^5^ the motion-corrected fMRI time series were averaged together and for each voxel, the aggregate receptive field properties of the underlying neuronal population were estimated using a 2D-Gaussian pRF-model. The model is described by three stimulus-referred parameters; pRF-center or the position preferred in the VF (*x* and *y* in Cartesian coordinates) and the spatial spread (σ). The time course of the stimulus is convolved with a canonical hemodynamic response function (HRF; Friston et al., 1998) to predict a voxel’s fMRI response. Approximately 100,000 predictions were generated for different plausible combinations of pRF parameters (*x*, *y*, σ) and the optimal pRF parameters, best fitting the predicted and actual voxel time-series were estimated by minimizing the sum of squared errors (RSS) between the two. Position parameters were used to compute voxel-wise eccentricity$\sqrt{(x^{2}y^{2}}$) and polar angle $\tan^{-1}\left(\frac{y}{x}\right)$ and the fitted 2D-Gaussian spatial spread was used to compute the pRF-size. For each participant, borders of the primary (V1) and extra-striate (V2 and V3) visual cortex were delineated by following the phase reversals in the polar angle data (Sereno et al., 1995) projected onto their inflated cortical surface.

##### Visual field coverage

We generated the coverage maps by back projecting the voxel-wise pRF estimates to a high resolution matrix (128 × 128) representing the VF. The coverage map shows the locations in the VF that elicit a significant response from the cortical voxels. Only voxels with an explained variance ≥15% were included for the generation of the VF-maps. The threshold was chosen based on existing literature (Baseler et al., 2011; Haak et al., 2012; Barton and Brewer, 2015). The pRF-center of each voxel along with its width (2D-Gaussian) was overlaid on the VF-matrix. In this way, each location in the VF might be represented by more than one pRF and the one with the maximum value was taken as the coverage measure at that specific location. The pRF coverage ranges between 0 and 1, where lower coverage values indicate a possible scotoma.

#### Block-Design fMRI

##### Visual stimulation and data analysis

Participants viewed a high-contrast pattern stimulus within a rectangular aperture [width: 48°; height: 28°] drifting in eight different directions, while maintaining fixation on a centrally located fixation dot. fMRI data were obtained during (a) passive viewing (PV) and (b) one-back-task (OBT; reporting the succession of identical motion directions) of the stimulus. In the controls, we simulated an artificial peripheral scotoma exposing only the central 7° of the stimulus through a circular aperture. The temporal sequence of each run followed a block design with 10 cycles of 12 s motion block (stimulus presentation) alternating with 12 s of mean luminance gray (24 s per cycle). Within each motion block, the direction of the contrast pattern was randomly changed every second (i.e., 12 trials per block). In each 1 s trial, the stimulus was presented for 750 ms followed by a 250 ms mean luminance gray. This fMRI data-set was analyzed previously for the assessment of task-dependences of the fMRI responses (Prabhakaran et al., 2021). Since, we use the results from these data exclusively for the selection of voxels for the VF-reconstruction analysis of the present study, we refer to the publication for details on preprocessing and analysis steps. Briefly, fMRI BOLD responses for the two conditions were quantified via voxel-wise phase specified coherence at the stimulation frequency [coherence*_*ps*_* (Masuda et al., 2008)].

##### Visual field reconstruction

For our non-mapping based VF-reconstruction a two-step strategy was employed, i.e., as first step we extracted pRF estimates from the retinotopic atlas for the voxels activated by the fMRI stimulus, as second step we reconstructed the VF based on these estimates. Specifically, we extracted the voxel coordinates which will be used for generating the VF-coverage maps from the fMRI data [threshold: coherence*_*ps*_* ≥ 0.30; *p* < 0.001 (uncorrected) (Silver et al., 2005)] and applied pRF-estimates from an atlas-defined retinotopic template to these voxels (Benson et al., 2014). The atlas has previously been demonstrated to predict the retinotopic organization of the visual cortex with high accuracy using only a participant’s brain anatomy. The anatomical retinotopic template is based on fMRI-based retinotopic mapping data and T1-weighted anatomy from 25 healthy participants as detailed in Benson et al. (2014). For the application of this template to the data-sets of the present study, a 3D cortical surface was generated from the anatomy of each participant and is inflated and flattened to a 2D surface. The patterns of the gyral and sulcal curvatures are used to register the 2D cortical surface between participants. Based on algebraic functions describing the topographic organization of the visual cortex (Schira et al., 2010), positions in the VF are mapped to points in the cortical surface. This algebraic retinotopic model is registered to the aggregate functional imaging data across the participants to construct the anatomical retinotopic template. With the voxel-wise pRF estimates from the template, we generated the VF-coverage maps applying the same procedure that was employed with the pRF mapping data. Separate coverage maps were computed for PV and OBT, respectively.

In addition, a Bayesian adaptation of the atlas-based approach (Benson and Winawer, 2018), i.e., combining participant-specific pRF-data with the retinotopic atlas, was also evaluated (here termed “Combined”). Coverage maps were generated similarly to the atlas-only approach.

### Quantification of Correspondence

On a participant-to-participant basis, the MD samples located in the central 14° of the SAP VFs were upsampled to match the spatial resolution [128 × 128] of the fMRI-derived coverage maps for a quantitative comparison. Subsequently, the coverage maps were binarized into responsive and non-responsive locations for the detection of absolute scotomas (threshold: MD −26 dB, i.e., sensitivity < 0 5dB). Similarly, fMRI-based VF-coverage maps were thresholded at a value of 0.7, in accordance with Ritter et al. (2019) for better comparability. Exploratory analysis with other threshold values below and above 0.7 resulted in suboptimal correspondence. VF-locations corresponding to the blind spot were not included in the analysis.

The primary correspondence between SAP and fMRI-based VFs was determined as in Ritter et al. (2019) and is defined by:

$$A\,c\,c\,u\,r\,a\,c\,y=\frac{\mathrm{Number}\,\mathrm{of}\,\mathrm{matched}\,\mathrm{VF}\,\mathrm{locations}\,\left(\mathrm{fMRI}\,\text{and}\;\mathrm{SAP}\right)}{\mathrm{Total}\,\mathrm{number}\,\mathrm{of}\,\mathrm{VF}\,\mathrm{locations}\,\mathrm{tested}}$$

The range of *Accuracy* is between 0 and 1, with higher values indicating a better agreement between the compared coverage maps.

For further exploratory evaluation, we also computed the sensitivity and specificity of fMRI for the scotoma detection as auxiliary measures.

$$S\,e\,n\,s\,i\,t\,i\,v\,i\,t\,y=\frac{\begin{array}{c} \mathrm{Number}\,\mathrm{of}\,\mathrm{matched}\,\mathrm{non}\,\mathrm{responsive}\,\mathrm{locations}\\ \left(\mathrm{fMRIand}\,\mathrm{SAP}\right) \end{array}}{\mathrm{Number}\,\mathrm{of}\,\mathrm{non}\,\mathrm{responsive}\,\mathrm{locations}\,\left(\mathrm{SAP}\right)}$$

$$S\,p\,e\,c\,i\,f\,i\,c\,t\,y=\frac{\begin{array}{c} \mathrm{Number}\,\mathrm{of}\,\mathrm{matched}\,\mathrm{responsive}\,\mathrm{locations}\\ \left(\mathrm{fMRIand}\,\mathrm{SAP}\right) \end{array}}{\mathrm{Number}\,\mathrm{of}\,\mathrm{responsive}\,\mathrm{locations}\,\left(\mathrm{SAP}\right)}$$

### Statistical Analysis

Data for the statistical analysis were prepared in MATLAB (MathWorks, Natick, MA, United States) and statistics were performed with the software “R,” version 3.4.1. Shapiro Wilk’s test was used to test the normality assumptions of the data and an appropriate test was chosen based on its outcome. For within group statistics, one-sample *t*-test of the differences between measures, conditions or approaches were used and for between-group statistics two-sample independent *t*-tests were employed. It should be noted that the statistics for the additional auxiliary measures, i.e., sensitivity and specificity, were not corrected for multiple comparisons, due to their exploratory nature.

## Results

In patients with advanced peripheral VF defects and controls with artificial scotomas, we investigated the scope of fMRI as an objective tool for VF-predictions. In a comparative approach with SAP-derived VFs, we determined the accuracy of different fMRI-based VF-reconstruction approaches [based on (1) pRF-mapping; (2) participant-specific anatomy driven retinotopic atlas for PV; and (3) OBT] and assessed the association of the fMRI-SAP correspondence with clinical characteristics.

### Cortical Representation of the VF-Defects

**FIGURE 1:**
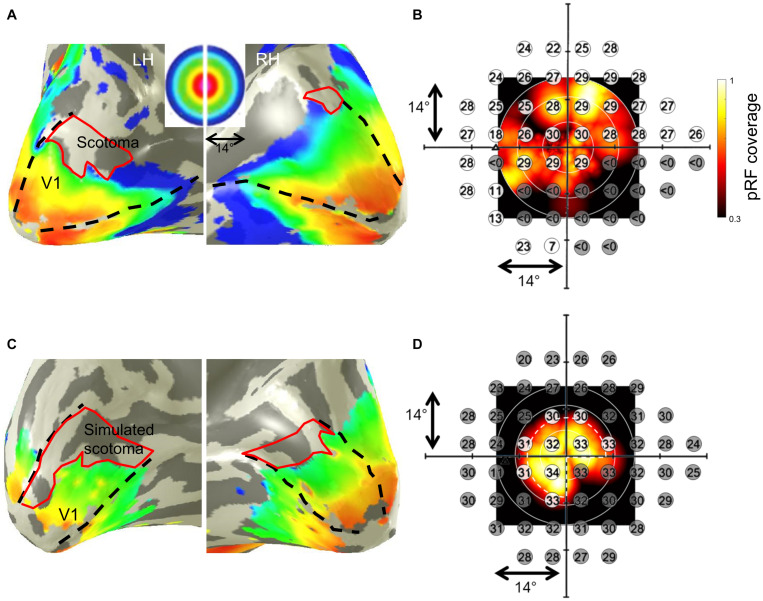
Population receptive field (pRF) mapping derived eccentricities of a representative glaucoma participant (GL1; stimulation of left eye) with lower VF-defect (scotoma) **(A)** and a control (HC5; stimulation of left eye) with simulated peripheral (>7°) and lower right quadrant scotoma **(C)** mapped on their subjective inflated right and left visual cortex, respectively. Dashed black lines in panels **(A,C)** illustrate the primary visual cortex V1 boundaries. False-color representation from dark orange to dark blue illustrates the retinotopic progression of eccentricities from 0° to 14°. Restricted representations are visualized in the regions of the cortex corresponding to the VF-deficits (depicted by the red borders). Panels **(B,D)** show the subjective VFs predicted by SAP overlaid on the pRF-based VF coverage map of the glaucoma patient and control, respectively. In panel **(B)**, sensitivity < 0 dB (indicated by gray discs) indicates absolute scotoma location in the glaucoma participant. In panel **(D)**, the white dashed pie represents the boundary of stimulated VF in the controls; the simulated scotomatous VF locations are indicated by the gray discs in the control. The superimposed plots clearly demonstrate the correspondence between the SAP-based and pRF-based VF-reconstructions for both, patient and control.

In all participants, we found a qualitative correspondence of the SAP-VF and the fMRI-based cortical VF maps. As an example of the cortical maps obtained, the eccentricity map derived from pRF mapping in a representative glaucoma participant (GL1) is depicted in Figure 1A. The maps clearly demonstrate a restricted representation in the anterior dorsal regions of the primary visual cortex (V1), which topographically corresponds to the lower peripheral VF defect of this participant. The superposition of the participant’s SAP-based VFs on the pRF-derived coverage maps (Figure 1B), demonstrates qualitatively the correspondence between the MRI and SAP-based VF-predictions. In controls with artificial scotoma, we report a similar correspondence between the two modalities, as depicted for a representative control participant in Figures 1C,D.

### Population Receptive Field-Based VF Reconstruction – Quantification of Agreement

#### How Does V1 pRF-Based VF Reconstruction Compare to Previous Reports?

We observed a strong correspondence between SAP-based and pRF-based VFs in our patient cohort (*n* = 6) with advanced peripheral deficits caused by glaucoma or RP [median accuracy (range): 0.62 (0.32–0.88)], which is similar to previous reports [e.g., RP patients in Ritter et al., 2019; accuracy (range): 0.85 (0.49–0.98)]. Remarkably, in our further separate evaluations of sensitivity and specificity in the patient cohort, we observed not only a high sensitivity of pRF-mapping in predicting VF-defects [median (range): 0.91 (0.74–1.0)], but also large false positive predictions of VF-defects [low specificity: 0.24 (0.11–0.99)]. We report a similar pattern in the sensitivity-specificity profile for our control cohort [accuracy: 0.74 (0.63–0.83); sensitivity: 1.0 (1.0–1.0); specificity: 0.41 (0.16–0.63)], which indicates this effect to be not patient-specific. Such an increase in false positives in the detection of VF-defects is likely associated with signal dropouts that are not exclusive to the regions of the visual cortex deprived of visual input, but that also affect the visually intact cortex (e.g., due to cortical folding patterns or venous anatomy), i.e., false-positive scotoma detection. Therefore, we investigated whether the low specificity arising from false-positive scotoma can be mitigated by pooling information from the early visual cortex (V1 through V3).

#### Does the Accuracy, Sensitivity, Specificity Benefit From Including V1–V3?

**FIGURE 2:**
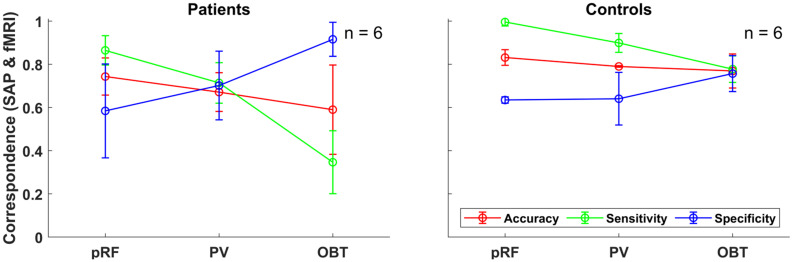
Group-level fMRI-SAP correspondence measures (accuracy; sensitivity; and specificity) of the early visual cortex (V1–V3-pooled) for the different fMRI approaches (pRF, PV, and OBT) visualized as line plots for the patients (*n* = 6) and controls (*n* = 6). Each data point and error bars represent the median of the corresponding measure and its median absolute deviation (MAD) around the median.

**FIGURE 3:**
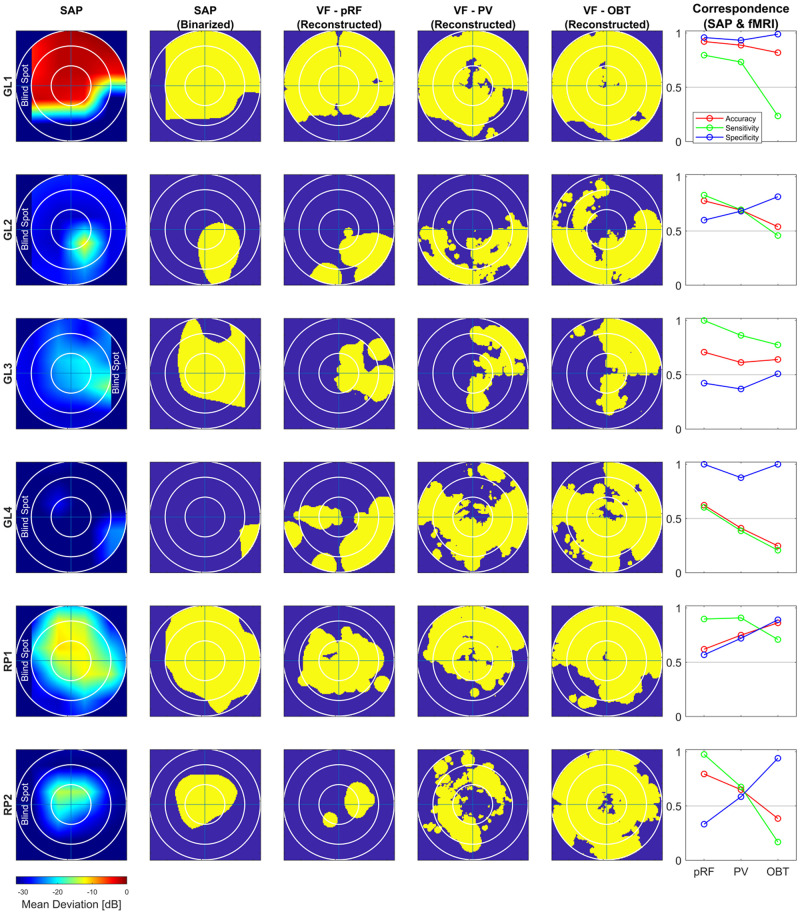
Comparison of subjective (SAP) and objective (fMRI) VF-reconstruction (V1–V3-pooled) in patients. Each row represents one participant, the description for the columnar subplots are as follows: SAP: Upsampled and interpolated SAP VFs for the central 14° (radius); SAP (Binarized): Binarization was performed by thresholding the SAP VFs into responsive (yellow) (MD ≤ –26) and non-responsive (scotoma) (MD > –26) locations; Columns VF – pRF, PV and OBT: Binarized pRF-based and atlas-based (PV and OBT) coverage maps, thresholded at a pRF coverage of 0.7; Correspondence (SAP and fMRI): accuracy; sensitivity; specificity for the different fMRI approaches (pRF, PV, and OBT) visualized as line plots.

**FIGURE 4:**
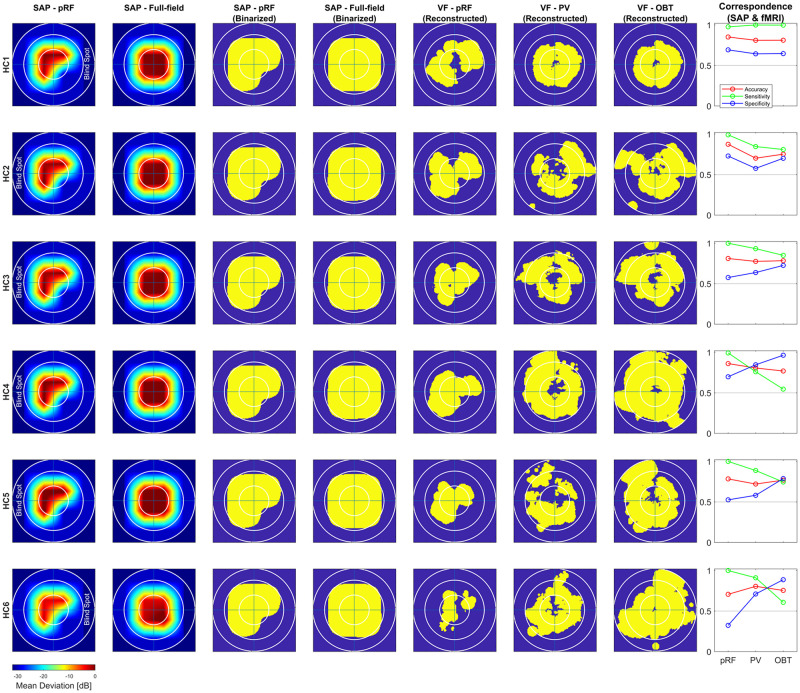
Comparison of subjective (SAP) and objective (fMRI) VF-reconstruction (V1–V3-pooled) in controls. Each row represents one participant, the description for the columnar subplots are as follows: Columns SAP-pRF and SAP-Full-field: Upsampled and interpolated SAP VFs for the central 14° (radius) for comparison with pRF-based and atlas-based coverage maps, respectively. The reason for two different SAP-VFs is, in addition to a peripheral artificial scotoma (>7°), the pRF-mapping stimulus had a quadrantopic scotoma stimulation as well. SAP-pRF and Full-field (Binarized): Binarization procedure for the two SAP-based VFs followed same convention as in Figure 3. Columns VF – pRF, PV, and OBT: Binarized pRF-based and atlas-based (PV and OBT) coverage maps, thresholded at a pRF coverage of 0.7; Correspondence (SAP and fMRI): conventions same as Figure 3.

To address the issue of asymmetric sensitivity-specificity profiles observed in the above V1-based VF reconstructions, we tested how accuracy, sensitivity and specificity measures for scotoma detection compare between V1–V3-pooled and V1-only data. In the patients, we observed as expected a trend to higher accuracies [median (range): 0.74 (0.62–0.92)] and an increased balance between the sensitivity [0.86 (0.6–1.0)] and specificity [0.58 (0.33–1.0)] than for the reconstructions based on V1-only [accuracy: 0.62 (0.32–0.88); sensitivity: 0.91 (0.74–1.0); specificity: 0.24 (0.11–0.99)]. In the controls with simulated scotoma an even higher correspondence of SAP and fMRI-based VF predictions was observed [accuracy: 0.83 (0.71–0.87); sensitivity: 1.0 (0.98–1.0); specificity: 0.63 (0.32–0.73)] compared to measures from V1-only [accuracy: 0.74 (0.63–0.83); sensitivity: 1.0 (1.0–1.0); specificity: 0.41 (0.16–0.63)] (see also Figure 2). The individual VF reconstructions are depicted in Figures 3, 4 for patients and controls, respectively. At the individual level, in comparison to V1-only metrics, a quantitative assessment of the VF-reconstructions for V1–V3-pooled data demonstrated a better correspondence accuracy between SAP and pRF-based VFs for 5/6 patients (exception: GL4; *t*(5) = 1.3, *p* = 0.255) and for all (6/6) controls [*t*(5) = 3.5, *p* = 0.017]. Taken together, there was a trend to increased accuracies for V1–V3 based VF-reconstructions, which reached significance in the controls. Consequently, we performed all subsequent analyses for the V1-V3-pooled data.

### Atlas-Based VF Reconstruction

Subsequent to the demonstration of a strong correspondence of pRF-based and SAP-based VFs, we investigated the feasibility of non-mapping based fMRI for VF predictions, as it has the potential to increase the utility and availability of fMRI-based objective VF-testing and its translation to clinical routine.

#### How Do pRF-Based and Atlas-Based VF Reconstructions of VF-Defects Compare?

In the patients, compared to mapping-based predictions [accuracy: 0.74 (0.62–0.92); sensitivity: 0.86 (0.6–1.0); specificity [0.58 (0.33–1.0)], we report for our anatomy informed retinotopic atlas and full-field stimulation (PV) approach equivalent accuracies [0.67 (0.41–0.89)] and a symmetric sensitivity [0.71 (0.39–0.91)] and specificity profile [0.7 (0.37–0.93] (Figure 2). In the controls, we observed very similar correspondence measures of the atlas-based (PV) and pRF-based approach [accuracy: 0.79 (0.7–0.81) vs. 0.83 (0.71–0.87); sensitivity: 0.9 (0.76–1.0) vs. 1.0 (0.98–1.0); specificity: 0.64 (0.58–0.85) vs. 0.63 (0.32–0.73)]. Only for the sensitivity measure, a significant decrease at the individual level for PV was found in both patients [*t*(5) = 3.2, *p* = 0.025] and controls [*t*(5) = 3.1, *p* = 0.026], which might be a consequence of the difference in the stimulation schemes between the two approaches. Taken together, the findings demonstrate the anatomy-based VF reconstructions with non-mapping full-field stimulation fMRI to be highly comparable to pRF-mapping based VF reconstructions [see Figures 3 (patients) and 4 (controls) for participant-specific atlas-based coverage maps and correspondence measures].

#### Benefits From Combined pRF- and Atlas-Based VF-Reconstruction?

In our additional analysis with combined pRF- and atlas-based data (Benson and Winawer, 2018) compared to the conventional atlas-based approach, we only observed marginal non-significant differences in the reconstruction accuracy [patients (combined vs. atlas-based): 0.74 (0.34–0.84) vs. 0.67 (0.41–0.89); controls: 0.77 (0.7–0.8) vs. 0.79 (0.7–0.81)]. However, a small but significant increase in the specificity with the combined approach was reported for patients [0.75 (0.42–0.94) vs. 0.7 (0.37–0.93; *t*(5) = 3.5, *p* = 0.017] and controls [0.77 (0.58–0.89) vs. 0.64 (0.58–0.85); *t*(5) = 3.4, *p* = 0.018], which is suggestive of potential benefits from a combined pRF-mapping and atlas-based approach, subject to the availability of mapping data.

#### Influence of Stimulus-Related Task (OBT) on Atlas-Based VF-Reconstruction?

We tested the informative value of the atlas-based VF-reconstruction approach and the three different performance measures (accuracy, sensitivity, and specificity) further. For this purpose, we applied the approach, in addition to the PV condition of the full-field stimulus, to the experimental condition OBT, which renders the cortical signature of the VF-defects in patients, but not controls, less specific (Masuda et al., 2010, 2008; Prabhakaran et al., 2021). For a meaningful measure, it is expected that the correspondence measures between SAP and atlas-based VF-reconstruction change for OBT compared to PV in patients, but not in controls.

**FIGURE 5:**
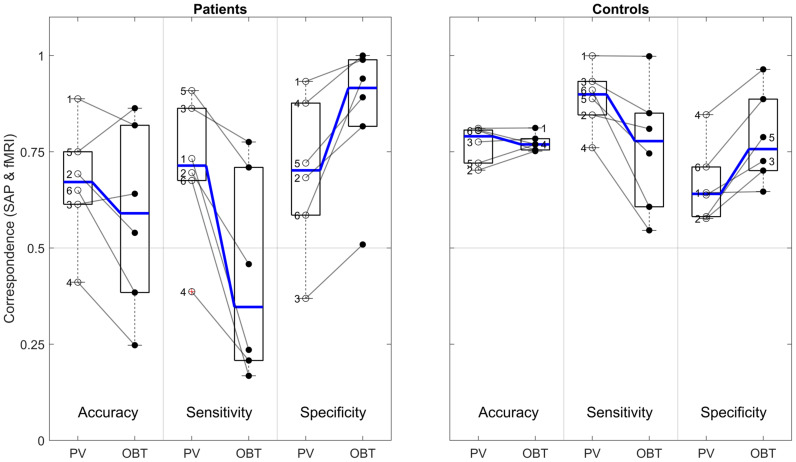
Difference in atlas-based correspondence measures (V1–V3-pooled) for PV and OBT in patients (left panel) and controls (right panel). Subpanels delineated by transparent gray lines encompass the boxplots for PV (left) and OBT (right) for accuracy, sensitivity and specificity, respectively. Thick horizontal blue lines within the box plot represent the median of the measure and the blue lines connecting the PV and OBT box plots indicates the slope of median difference between them. Participant-specific correspondence measures, superimposed on the box plots (PV – open dots and OBT – closed black dots) are connected by a line to indicate the slope of the difference. Numbers 1–4 in the patients’ panel represents the glaucoma participants GL1–GL4 and 5 and 6 represent RP1 and RP2. In the controls panel, 1–6 represents HC1–HC6, respectively.

Remarkably, for the patients’ OBT, we found accuracies [0.59 (0.25–0.86)] closely similar to PV [0.67 (0.41–0.89); *t*(5) = 1.5, *p* = 0.192]. In contrast, the expected reduced performance for OBT was evident for sensitivities [median sensitivity for PV and OBT (range): 0.71 (0.39–0.91) and 0.35 (0.17–0.78), respectively; *t*(5) = 4.0, *p* = 0.011] and specificities [PV: 0.70 (0.37–0.93); OBT: 0.92 (0.51–1.0), respectively; *t*(5) = −3.9, *p* = 0.011]. Figure 5 demonstrates that, at the individual level, all patients showed a considerable decrease in sensitivity and an increase in specificity of the OBT driven atlas-based approach. As anticipated, in controls with a simulated peripheral scotoma, a difference in the accuracy [PV: 0.79 (0.7–0.81); OBT: 0.77 (0.75–0.81); *t*(5) = −0.2, *p* = 0.851] was not observed. Unexpectedly, we did find a significant difference in the sensitivity [PV: 0.9 (0.76–1.0); OBT: 0.78 (0.55–1.0); *t*(5) = 2.7, *p* = 0.041] and specificity [PV: 0.64 (0.58–0.85); OBT: 0.76 (0.65–0.96); *t*(5) = −4.1, *p* = 0.010], similar to the patients. However, a further exploratory two-sample independent *t*-test (patients > controls) demonstrated the patients to have a greater magnitude of PV-OBT difference than controls [sensitivity (*t*(10) = 1.8, *p* = 0.05); specificity (*t*(10) = 2.1, *p* = 0.031)]. Nevertheless, from our observations for PV and OBT, task-dependent dynamics in the correspondence measures is noticeable in both patient and controls. This suggests a simple full-field stimulus without an explicit task to be the optimal choice for atlas-based VF-reconstruction approaches.

### Correlation With Clinical Characteristics

**FIGURE 6:**
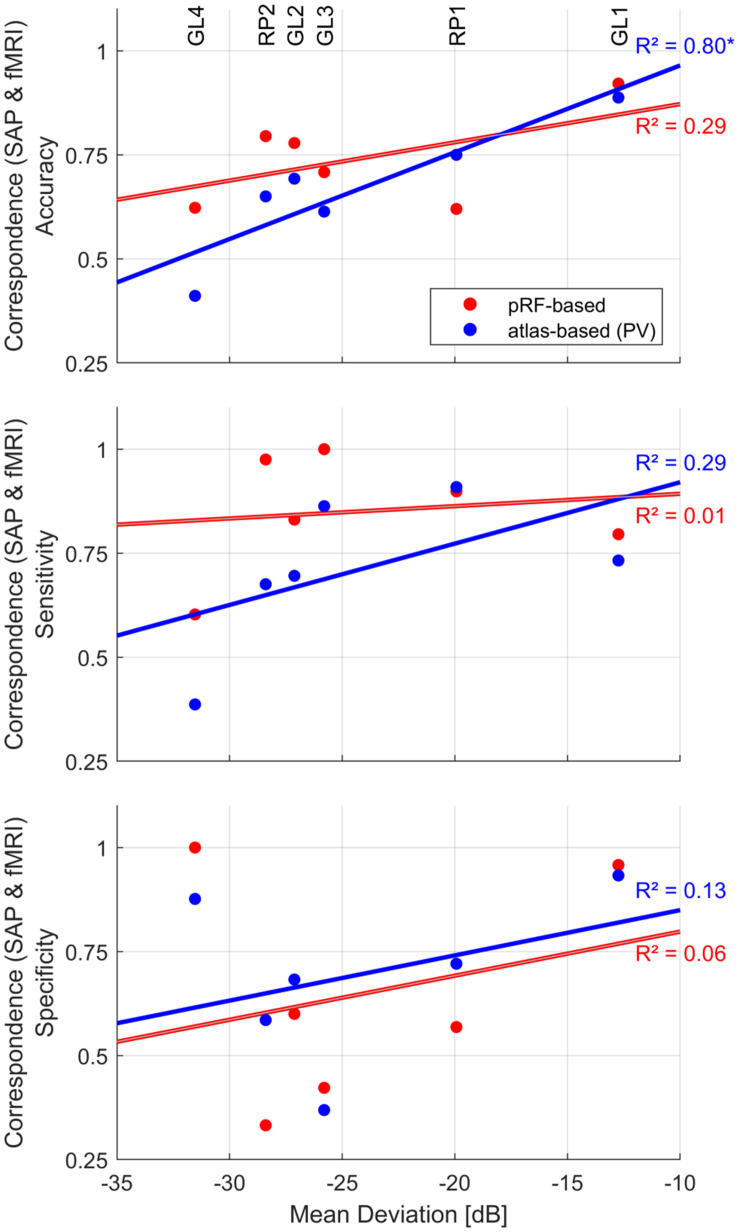
Dependence of fMRI-SAP correspondence measures [pRF-based (red) and atlas-based PV (blue)] on mean deviation (MD) of the central 14° VF. Patient-specific measures are plotted as scatter-dot plots. Solid lines depict the best fitting regression. The strength of the association was evaluated as coefficient of determination (linear regression model); “*” indicates a significant *p*-value.

Insights into the association of fMRI-based VF predictions with patient-specific clinical characteristics are critical for its translation to clinical routine. Therefore, we investigated this relationship in the patients of the present study. Specifically, we explored the dependence of the correspondence measures on the MD as predicted by SAP (Figure 6) using a simple linear regression model [*R*^2^ (coefficient of determination)]. All analyses were confined to the central 14° VF. For the atlas-based approach (PV), we observed a strong significant linear relationship between fMRI reconstruction accuracy and MD [*R*^2^ = 0.80, *p* = 0.014]. This did not apply to the pRF-based approach [*R*^2^ = 0.29, *p* = 0.796]. There was no significant association for sensitivity and specificity, irrespective of the reconstruction approach.

## Discussion

In the present study, we investigated for a group of patients with advanced peripheral VF-deficits (glaucoma and RP) and for HCs with simulated peripheral scotoma, the potential of various fMRI-based approaches for the reconstruction of VFs. We report a strong correspondence between the SAP-based and pRF-mapping-based VF reconstructions especially for pooled data from V1-V3. Equivalent correspondences were observed for VF-reconstructions that were based on simple block-design full-field stimulation fMRI data combined with an individualized anatomy-driven retinotopic atlas. In addition to our primary metric of correspondence, i.e., correspondence accuracy, we found the use of supplementary metrics to assess VF-defect prediction, i.e., sensitivity and specificity, to be critical to pinpoint and understand factors that might be of influence on the quality of fMRI-based reconstructions.

Qualitatively, the cortical response signatures observed in our patients corresponded to the location of their VF-defects, which is in accordance with the well-established application of retinotopic fMRI in mapping retinal lesions in the visual cortex (Duncan et al., 2007; Baseler et al., 2011; Barton and Brewer, 2015; Ferreira et al., 2017). Our finding of a moderate quantitative correspondence accuracy between SAP- and pRF-based VFs from V1-only data are in line with previous reports (Papanikolaou et al., 2014; Silson et al., 2018; Ritter et al., 2019; Carvalho et al., 2021). This prompts the question, why the correspondence of SAP and pRF-based VFs is not higher. We would like to indicate three potential reasons for this observation. (i) Cross-modality comparison. The comparison is done between two modalities, SAP vs. fMRI, that are fundamentally different, in terms of the entire approach, i.e., threshold detection of a spot-light vs. cortical responses to a temporally modulated high contrast checker-board exposed through a bar sweeping across the screen. (ii) Different sampling of the VF. For fMRI-based VF reconstruction, the VF is sampled much more densely than for SAP (one data point covered 6° × 6°). As a consequence, the SAP-results were upsampled for the comparison with fMRI-VFs, which likely contributed to a mismatch in the inter-modal comparison. (iii) Correspondence metric. The add-on metric of specificity indicated fMRI susceptibility to false-positive detection of VF-defects, i.e., overestimation of the scotoma, to be a critical factor in determining the correspondence. The proportion of false-positives was observed to follow an inverse relationship with the extent of the VF-defects. In fact, this is plausible, as an individual with a very large scotoma would have fewer responsive locations to be mislabeled as non-responsive.

As we report these false-positive detections even in the controls, we reason the cause to be of methodological origin rather than physiological, for e.g., signal dropouts as a result of reduced modulation of cortical responses or morphological limitations as in venous anatomy or cortical folding patterns generating local magnetic field inhomogeneities. This is complemented by our observations of reduced false-positive scotoma detection and consequent increase in accuracy with pooling of V1–V3 mapping-data for the reconstruction. Pooling V1–V3 appears to help in covering the VF-locations with signal dropouts for V1-only data. Considering V2 and V3 receive their primary input from V1 neurons, a potential logic for the observed effect of pooling might be that the neurons in a voxel associated with signal dropout may still drive voxels in V2 and V3. Thus pooling data from the three visual areas increases the likelihood of an fMRI response from at least one of the areas thereby contributing to the VF. However, the smaller surface area of V2 and V3 retinotopic maps in comparison to V1’s and consequent coarse sampling might result in less precise and crude VF maps with the pooled data. Moreover, the increase in pRF sizes along the visual hierarchy might also add-up to this imprecision. Taken together, it should be noted that while pooling V1-V3 might ameliorate the incidence of false positives, it may also limit the ability to detect small scotomas due to a filling-in type of effect. Nevertheless, identifying the exact mechanisms behind the reported increase in accuracy of correspondence with pooled data is beyond the scope of this study and warrants future research, as information on VF-predictions based on individual visual areas are critical for establishing fMRIs likely role in therapeutic decisions.

Recent promising advancements in cell-, gene-, and microelectronics based vision restoration procedures (Ashtari et al., 2015; Aguirre, 2017; Roska and Sahel, 2018; Beauchamp et al., 2020) led to an increased fundamental interest in fMRI as a tool for objective visual function assessment. These upcoming therapeutic interventions require precise information of the VF representation in the visual cortex following VF-loss, which is provided by mapping-based fMRI. A bottleneck, however, is acquiring this information in patients where fMRI-based mapping is not feasible, for instance due to unstable fixation, very advanced VF loss or inability to comply with demanding task requirements. The VF-reconstruction approach employed here, using simple fMRI stimulus driven cortical responses in combination with an individualized retinotopic atlas demonstrated a performance that is equivalent to the pRF-based approach. The utility of this atlas-based approach also finds support from a previous report on two patients with Leber congenital amaurosis to investigate changes in fixation location (pseudo fovea) pre and post retinal gene therapy (Cideciyan et al., 2015). The stimulus used by Cideciyan and colleagues was a flickering uniform luminance screen whereas we employed a high contrast moving grating stimulus. Technically, the approach is expected to be robust to the use of any simple and salient stimulus, nevertheless it would be of interest for future work to test for any stimulus-type dependent effects on the approaches VF-reconstruction capability.

The use of spatially specific stimuli for pRF-mapping makes the approach susceptible to eye movements (Hummer et al., 2016). The full-field stimulus used in the atlas-based approach has the advantage to be less sensitive to fixation instabilities. Although in our experiment the participants were presented with a fixation dot and instructed to focus their attention, it should, in fact be possible to discard the fixation and apply a free-viewing approach to the stimulus. This was not achievable with the current setup of our fMRI visual stimulation system which had a limited stimulus window size [width × height: 48° × 28°] and this limitation could be overcome by the use of wide-field stimulus displays (Wu et al., 2013; Greco et al., 2016).

We found a significantly reduced sensitivity for the detection of VF-defects with the atlas-based approach, when a stimulus-related task (OBT) was introduced. This indicates that the quality of VF-reconstructions is task-dependent and reduced if attention is directed to the visual stimulus. While this is at first sight counter-intuitive finding, it corresponds well with earlier reports on patients with central and peripheral VF deficits, where a stimulus-related tasks drove responses in the deafferented regions of the visual cortex (Baker et al., 2008; Masuda et al., 2010, 2008; Ferreira et al., 2019). The origin of these task-dependent responses is still under debate and beyond the scope of this study, for the purpose of atlas-based VF assessments. Still we can draw an important conclusion from our current findings, i.e., that including a stimulus-related attention task is, counter-intuitively, not recommended as it induces unspecific activations in deafferented cortex. It should be noted, however, that we here tested for effects of global attention as opposed to spatially varying attention. Consequently, it is unknown, whether there would be any differential effects of spatially-specific attention to the stimulus-aperture, e.g., in the pRF-stimulation sequence. It is to be noted that even in the absence of a task (PV) we did observe a marginal, but significant decrease in the sensitivity compared to pRF-based reconstruction. There might be two reasons for this, (1) the distinction between the pRF-mapping and PV stimulus by itself might drive the cortex differentially, and (2) participants performing OBT subconsciously even during PV, as the instructions for both PV and OBT were given pre-scanning. Nevertheless, our data show that the stimulus used in the atlas-based (PV) reconstruction performs equivalently well as the mapping-based approach in reconstructing VFs. This suggests that a simple block design stimulus without an explicit task is the optimal choice.

We acknowledge the small sample size, which was still sufficient for a statistical inference of the results. As we included patients with very advanced VF-defects, most of the recruited patients were aged and consequently resulted in a high rate of exclusions due to at least one MRI-related contraindication. The small sample size also limits our ability to correlate the performance of fMRI-based VFs with patient-specific clinical characteristics, when in fact a linear trend was observed with MD from SAP. Information on the relationship with clinical correlates is critical for translation of fMRI to clinical routine, which must be addressed by future research with patients with different stages of pathology using wide-field stimulation approaches.

In studies with patients who are prone to suffer from unreliable fixation, for instance, as a result of low visual acuity or large VF defects, the availability of quantitative eye-tracking data adds validation to the inference of results. While some of our patients fall in the aforementioned category, all of them were able to fixate quite well (fixation stability for the central 2° radius > 96%), as determined with fundus-controlled perimetry and a qualitative monitoring of stimulated eye in the scanner using an eye-tracker. This was also evident from their ability to perform a fixation dot task for the pRF-mapping experiment, subsequently confirmed by an overall good quality of retinotopic maps. Nevertheless, the lack of quantitative eye-tracking data should still be considered a constraint and we underscore the importance of eye-tracking in studies involving patients with vision disorders.

Although other mapping-based fMRI approaches, as in temporal phase-encoding (conventional rings and wedges) have also been employed in mapping VF defects in patients (Morland et al., 2001; Furuta et al., 2009; DeYoe et al., 2015), due to the prevalent adoption of pRF-mapping in recent years, we chose the latter approach for VF-mapping here. A few important similarities and differences with these approaches should be noted. (1) The stimulus used for both the pRF-mapping and phase-encoding methods are spatially-selective and suffer from the same limitations of requirement for stable fixation and attention from the patients. (2) In contrast to the phase-encoding method, the model-based analysis of pRF-mapping data provides a direct estimation of neuronal receptive field size (pRF-size) and this information is expected to enhance the accuracy of the reconstructed VFs. (3) pRF-mapping data provides precise VF-maps to the center of the foveal representation (Dumoulin and Wandell, 2008). (4) Although the acquisition time for these approaches are quite similar, analysis of conventional mapping data is less time-consuming. In consideration to the above-mentioned pros and cons, we believe a critical discussion on the situation-dependent suitability of the methods might help in making an informed decision on the choice of the mapping technique. For example, for the purpose of a time-constraint surgical planning which might not require a highly precise VF-map, fMRI-reconstruction based on phase-encoding approach might suffice.

The anatomy driven retinotopic atlas used in the atlas-based approach is based on pRF-mapping data from HCs and could be argued as a bias when used in patients with VF-defects. This could be asserted in consideration to studies that report altered pRF properties (specifically shifting of pRF position and enlargement of pRFs) in such patients (Ferreira et al., 2017; Zhou et al., 2017) and suggestive of cortical reorganization. It is to be noted, however, there is no clear consensus on this as there is a growing body of evidence demonstrating similar changes in receptive field properties even in controls with simulated scotomas (Baseler et al., 2011; Haak et al., 2012; Prabhakaran et al., 2020). Ideally, resolving this would require the creation of separate atlases specific for the patient population, but given the heterogeneity manifested in vision disorders it seems to be far-fetched at this point of time. Taking into account, the limited scope of long-term reorganization of the adult visual cortex in acquired vision disorders (Wandell and Smirnakis, 2009), we do not see the use of a control-based atlas as a potential limitation in the study.

Finally, it should be acknowledged that in the present study the atlas-based reconstruction of VFs is based on the assumptions of undistorted central representation and absence of retinotopic re-organization. This might limit the method’s utility to acquired peripheral vision disorders. Considering this, based on our current data and results, we exercise caution and warrant future research to investigate the applicability of the approach to: (1) central vision disorders (e.g., macular degeneration) even though with pRF-mapping being previously demonstrated to be a feasible tool to map central VF-defects (Hummer et al., 2018; Ritter et al., 2019), (2) congenital vision disorders with possible reorganization (Baseler et al., 2002), and (3) pediatric and very young individuals who would still be in the developmental phase of their brain anatomy.

## Conclusion

In summary, we demonstrated in patients with advanced peripheral VF-defects (glaucoma and RP) and in controls with simulated scotomas the feasibility of fMRI as a tool for objective assessment of VFs. We report a good agreement between the VFs predicted by pRF-mapping and SAP, which is consistent with existing reports, thereby affirming the reliability of the technique. Importantly, we observed the atlas-based approach with a full-field simple block design stimulus perform equally well in reconstructing VFs based on cortical responses. Consequently, the results serve as a proof of concept for the atlas-based procedure to be a surrogate fMRI method in the absence of mapping data and to be of substantial benefit in studies involving patients with peripheral VF-defects. These findings are expected to provide guidance to overcome current limitations of translating fMRI-based methods to a clinical work-up.

## Data Availability Statement

The raw data supporting the conclusions of this article will be made available by the authors, without undue reservation.

## Ethics Statement

The studies involving human participants were reviewed and approved by ethics committee – the University of Magdeburg. The patients/participants provided their written informed consent to participate in this study.

## Author Contributions

GP: conceptualization, methodology, formal analysis, investigation, data curation, and writing – original draft. MH: conceptualization, methodology, supervision, writing – review and editing, and funding acquisition. KA-N: investigation, writing – review, and editing. CT: methodology, writing – review, and editing. HT: writing – review and editing. All authors contributed to the article and approved the submitted version.

## Conflict of Interest

The authors declare that the research was conducted in the absence of any commercial or financial relationships that could be construed as a potential conflict of interest.

## Publisher’s Note

All claims expressed in this article are solely those of the authors and do not necessarily represent those of their affiliated organizations, or those of the publisher, the editors and the reviewers. Any product that may be evaluated in this article, or claim that may be made by its manufacturer, is not guaranteed or endorsed by the publisher.

## Footnotes

1
https://surfer.nmr.mgh.harvard.edu/

2
https://github.com/vistalab/itkgray

3
https://afni.nimh.nih.gov/

4
https://github.com/kendrickkay/alignvolumedata

5
https://github.com/vistalab/vistasoft
